# PAR2-Induced Tissue Factor Synthesis by Primary Cultures of Human Kidney Tubular Epithelial Cells Is Modified by Glucose Availability

**DOI:** 10.3390/ijms22147532

**Published:** 2021-07-14

**Authors:** Tyrone L. R. Humphries, Kunyu Shen, Abishek Iyer, David W. Johnson, Glenda C. Gobe, David Nikolic-Paterson, David P. Fairlie, David A. Vesey

**Affiliations:** 1 Centre for Kidney Disease Research, Translational Research Institute, Faulty of Medicine, The University of Queensland at the Princess Alexandra, Brisbane, QLD 4072, Australia; t.humphries@uq.edu.au (T.L.R.H.); kunyushen94@gmail.com (K.S.); david.johnson2@health.qld.gov.au (D.W.J.); g.gobe@uq.edu.au (G.C.G.); 2 Australian Research Council Centre of Excellence in Advanced Molecular Imaging, Institute for Molecular Bioscience, The University of Queensland, Brisbane, QLD 4072, Australia; a.iyer@uq.edu.au (A.I.); d.fairlie@uq.edu.au (D.P.F.); 3 Centre for Inflammation and Disease Research, Institute for Molecular Bioscience, The University of Queensland, Brisbane, QLD 4072, Australia; 4 Department of Nephrology, The University of Queensland at Princess Alexandra Hospital, Brisbane, QLD 4102, Australia; 5 School of Biomedical Sciences, Faculty of Medicine, The University of Queensland at the Translational Research Institute, Brisbane, QLD 4072, Australia; 6 Department of Nephrology, Monash Medical Centre and Monash University Centre for Inflammatory Diseases, Melbourne, VIC 3168, Australia; david.nikolic-paterson@monash.edu

**Keywords:** protease, glucose, PAR2, tissue factor, kidney tubular epithelial cells, diabetes

## Abstract

Coagulopathies common to patients with diabetes and chronic kidney disease (CKD) are not fully understood. Fibrin deposits in the kidney suggest the local presence of clotting factors including tissue factor (TF). In this study, we investigated the effect of glucose availability on the synthesis of TF by cultured human kidney tubular epithelial cells (HTECs) in response to activation of protease-activated receptor 2 (PAR2). PAR2 activation by peptide 2f-LIGRLO-NH_2_ (2F, 2 µM) enhanced the synthesis and secretion of active TF (~45 kDa) which was blocked by a PAR2 antagonist (I-191). Treatment with 2F also significantly increased the consumption of glucose from the cell medium and lactate secretion. Culturing HTECs in 25 mM glucose enhanced TF synthesis and secretion over 5 mM glucose, while addition of 5 mM 2-deoxyglucose (2DOG) significantly decreased TF synthesis and reduced its molecular weight (~40 kDa). Blocking glycosylation with tunicamycin also reduced 2F-induced TF synthesis while reducing its molecular weight (~36 kDa). In conclusion, PAR2-induced TF synthesis in HTECs is enhanced by culture in high concentrations of glucose and suppressed by inhibiting either PAR2 activation (I-191), glycolysis (2DOG) or glycosylation (tunicamycin). These results may help explain how elevated concentrations of glucose promote clotting abnormities in diabetic kidney disease. The application of PAR2 antagonists to treat CKD should be investigated further.

## 1. Introduction

Diabetic kidney disease (DKD) develops in approximately 40% of diabetics and is the leading cause of chronic kidney disease (CKD) worldwide [1,2]. It is a major health care burden on the economy ($78 billion in US alone in 2016), and greatly increases the risk of developing hypertension and cardiovascular disease (CVD), conditions that often lead to death before kidney failure takes its toll [3,4]. Understanding the mechanism by which DKD develops and how it impacts CVD is vitally important to progressing new treatment strategies.

Patients with diabetes and CKD often have blood coagulation disorders [5,6,7,8] associated with enhanced synthesis and premature activation of the coagulation system components. This may present as thrombotic microangiopathy or disseminated intravascular coagulation [9,10,11]. In CKD, fibrinogen expression and fibrin deposits in the kidneys are common [7,8]. Tissue factor (TF), which plays a central role in initiating the injury-induced blood coagulation cascade, is a potential trigger of this fibrin deposition. TF is an integral transmembrane glycoprotein with a molecular weight of ~45 kDa (by immunoblotting) [12]. The extracellular N terminus has 219 amino acids residues and three potential glycosylation sites (Asn^11^, Asn^124^, and Asn^137^). The cytoplasmic C-terminal domain of 19 amino acids has one potential glycosylation site (Asn^261^) [12,13]. Controversy remains about the influence of post-translational glycosylation on TF pro-coagulant activity [14,15] but the molecular weight of TF does vary between tissue type and disease status in a manner consistent with differential glycosylation, which seemingly affects its functional properties as for other proteins [12,16].

TF is not normally expressed by cells exposed to blood, such as vascular endothelial cells, but is highly expressed by subendothelial cells. Injury to the blood vessels leads to TF exposure and rapid clot formation. TF is expressed by many other cell types, such as brain astrocytes, lung epithelial cells, placental endothelial cells, and heart myocytes [17]. TF, which lacks proteolytic activity of its own, is activated by a conformational change that leads to sequential activation of factor VII, factor X, thrombin, and cleavage of fibrinogen to fibrin. Low concentrations of TF are found in the circulation associated predominantly with microparticles (MPs) that appear to originate from monocytes/macrophages and/or injured subendothelial cells [17]. Elevated TF-positive MPs have been reported in a range of conditions including diabetes, dyslipidemia, and malignancies.

Protease-activated receptors (PARs) are protease-sensing G protein-coupled receptors widely expressed on the surfaces of cells throughout the body [18]. Four members (PAR1–4) have been identified and have been associated with epithelial barrier homeostasis, inflammation, pain, and metabolism. Protease-activated receptor-2 (PAR2) is activated by a range of serine proteases including coagulation-associated proteases, such as clotting factor VIIa, and factor Xa, trypsin and matriptase [18,19,20]. Cleavage of the N-terminal extracellular domain of PAR2 by a protease exposes a new N terminus, which binds to the body of the receptor to trigger activation and a cascade of intracellular signaling events including elevation of intracellular Ca^2+^, cyclic adenosine monophosphate (cAMP) and phosphorylated intracellular kinases, such as extracellular-signal-regulated kinase (ERK) and protein kinase B (AKT) [21]. In addition to proteases, synthetic peptides of six amino acids, such as 2-furoyl-LIGRLO-NH_2_ (2F), have been widely used as exogenous agonists to investigate roles for PAR2 in physiological and pathophysiological situations. Within the human kidney, PAR2 is especially prominent in the tubule epithelial cells of the kidney cortex and kidney vasculature and enhanced expression in these cells has been reported in various inflammatory kidney diseases [20,22,23].

Here, we investigate TF synthesis in HTECs in response to PAR2 activation when glucose availably is modified. We show that PAR2 activation in HTECs induces TF synthesis, which is enhanced by the elevated availability of glucose and reduced by inhibiting glycolysis and/or glycosylation or by a PAR2 antagonist.

## 2. Results

### 2.1. PAR2 Activation Induces Synthesis of Tissue Factor by HTECs

**Figure 1:**
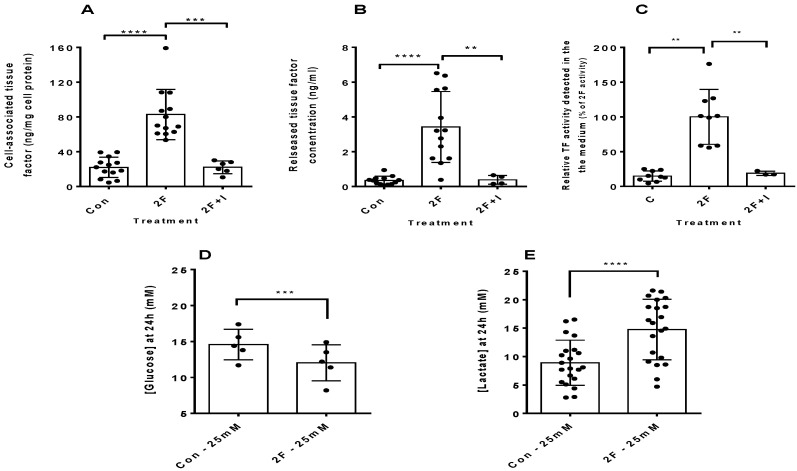
Activation of PAR2 by 2f-LIGRLO-NH_2_ (2F) induces tissue factor (TF) synthesis and secretion from HTECs. (**A**,**B**) Attenuation by a PAR2 antagonist, I-191 (I, 10 μM). 2F induces enhanced glucose consumption and lactate secretion. (**A**,**B**) TF-specific ELISA was used to measure the TF concentrations produced by HTECs treated with or without 2F. (**C**) A chromogenic factor Xa activity assay was used to assess the TF activity of conditioned medium (secreted TF). (**D**,**E**) The final glucose and lactate concentrations of the conditioned medium from control cells and 2F-treated cells. T-tests were used to determine significance of mean differences. Bars represent the mean ± SD. **, *** and **** indicate significance of *p* < 0.01, *p* < 0.001 and *p* < 0.0001, respectively. n ≥ 3 for all treatments. Con = Control, 2F = 2f-LIGRLO-NH_2_ (2 µM), I = I-191 (10 µM).

Both human and animal studies have shown fibrin deposition in various forms of kidney disease [7,17,24,25,26]. The mechanism of its production, however, is poorly understood. As the central initiator of the blood coagulation cascade, which ultimately results in fibrin deposition, a dysregulated expression of TF could be responsible. We found that PAR2 activation, in HTECs, with a potent synthetic PAR2 agonist peptide, 2F, induced enhanced synthesis and secretion of TF by 4-fold and 9-fold, respectively, over a 24 h experimental period (Figure 1A,B).

The amount of TF released into the medium of 2F-treated cells was less than 1% of the total TF produced. A potent PAR2 antagonist, I-191 (10 µM), was able to completely abrogate this TF synthesis and secretion. Conditioned medium from cells grown in 25 mM glucose and treated with 2F showed enhance ability to activate factor X in the TF activity assay (Figure 1C). We noticed that the medium of 2F-treated cells became yellowed much quicker than control-treated cells. Thus, we measured the level of lactate and glucose present in the conditioned medium at the end of the experiment. It was found that there was a significant increase in lactate production in the 2F-treated cells which was mirrored by an increased consumption of glucose (Figure 1D,E). Taken together, these results confirm that PAR2 activation specifically induces TF synthesis and that at least a small portion of this is released from cells in an active form. This increased TF synthesis is accompanied by an increased flux of glucose through glycolysis and enhanced production of lactate.

### 2.2. PAR2-Induced TF Synthesis by HTECs Is Upregulated by Glucose

**Figure 2:**
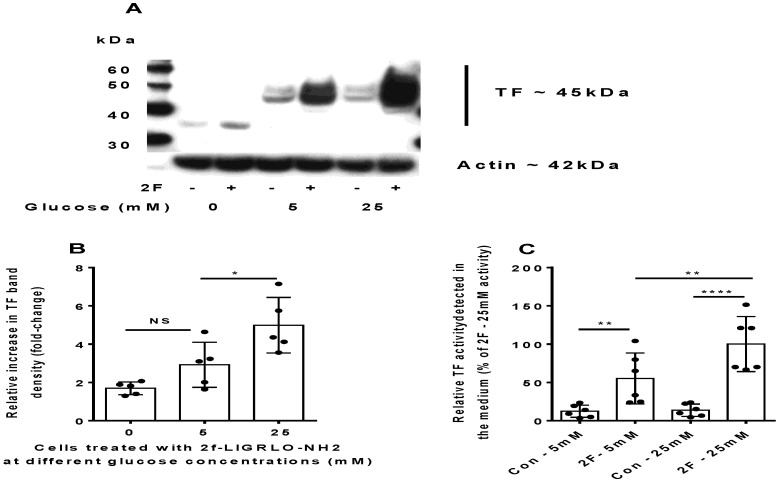
PAR2-induced TF synthesis and secretion by HTECs is enhanced by culture in a medium containing high glucose (25 mM) compared to low glucose (5 mM). (**A**) Representative Western blot showing the effect of glucose concentration on induction of tissue factor synthesis induced by 2F. (**B**) Semiquantitative analysis of the change in expression of TF-induced 2F when cells were cultured in 0, 5 or 25 mM glucose as measured by Western blot, (n = 5). (**C**) A chromogenic factor Xa activity assay was used to assess the TF activity present in the conditioned medium (secreted TF). (See Section 4 for details). T-tests were used to determine the significance of mean differences. Bars represent the mean ± SD *, **, and **** indicates significance of *p* < 0.05, *p* < 0.01 and *p* < 0.0001, respectively.

One of the hallmarks of diabetes is resistance of cells to the action of insulin. This translates into an inability to regulate plasma concentrations of glucose which are often elevated beyond physiologically normal levels, and this can lead to cell damage in multiple organ systems. Western blotting was used to measure the amount and molecular weight of TF produced by HTECs cultured in the presence of 5 and 25 mM glucose with or without PAR2 activation. TF was constitutively expressed by HTECs cultured in either 5 or 25 mM glucose, although the levels appeared to be relatively low. There was no significant change in TF expression by cells when cultured in medium containing 5 or 25 mM glucose alone. However, PAR2 activation led to an apparent 1.7-fold increase TF synthesis in medium containing 25 mM glucose than that containing 5 mM glucose (Figure 2A,B).

The molecular weight of TF produced remained at 45 kDa at both concentrations. In cells cultured without glucose (0 mM), however, the TF produced was predominantly of 36 kDa. Some fainter bands up to 45 kDa were also observed on further development of the blot and attributed to different levels of TF glycosylation when glucose was not available. A TF-activity assay revealed that active TF secretion was significantly greater when PAR2 was activated in the presence of 25 mM glucose than 5 mM glucose (Figure 2C). To exclude the possibility that 25 mM glucose induces TF by an osmotic effect, in some experiments, we include L-glucose to the concentration of D-glucose used. An equivalent concentration of L-glucose was unable to induce TF synthesis as D-glucose did (data not shown). Taken together, these results demonstrate that with high levels of available glucose, the HTECs can synthesize increased amounts of TF, some of which is secreted and active. Glucose availability appears to be required for TF glycosylation.

### 2.3. PAR2-Induced TF Synthesis Is Glucose Dependent

**Figure 3:**
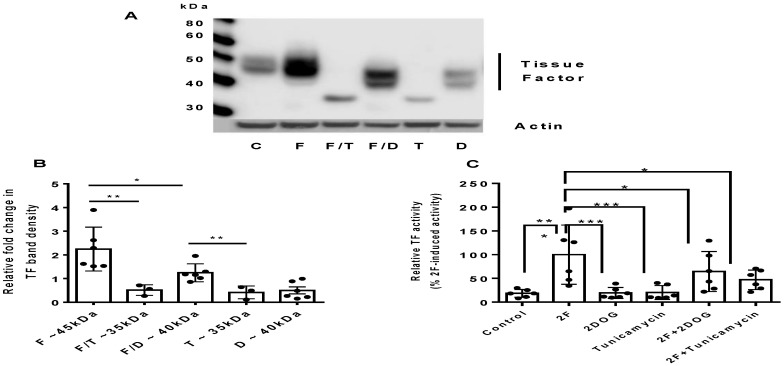
2-Deoxyglucose and tunicamycin reduce PAR2-induced TF synthesis and its molecular weight in HTECs. (**A**) Representative Western blot showing the effect of 2-deoxyglucose or tunicamycin based on amount and molecular weight of TF induced by 2F. (**B**) Semiquantitative analysis of the change in expression of TF induced by 2F when cells were cultured in the presence of 2DOG (5 mM) or tunicamycin (4 µg/mL) as measured by Western blot. (**C**) A chromogenic factor Xa activity assay was used to assess the TF activity of conditioned medium (secreted TF). Cells were treated with or without 2F (2 μM) in the presence of either 2-deoxyglucose (5 mM) or tunicamycin (4 μg/mL). The experiment was repeated ≥3 times with different donor cells. The bars represent the mean ± SD. A significant * (*p* < 0.05), ** (*p* < 0.01), *** (*p* < 0.001) increase in TF synthesis compared to vehicle treatment (control). C = control (vehicle), F = 2f-LIGRLO-NH2, (2 μM), D = 2-deoxyglucose (5 mM), and T = tunicamycin (4 mg/mL).

As glucose enhanced PAR2-induced TF synthesis, we investigated further whether it was modulated by disrupting glycolysis. We found that blocking glycolysis with 2-deoxy-D-glucose (2DOG; 5 mM) in the presence of a 25 mM D-glucose significantly reduced PAR2-induced TF synthesis (Figure 3A). Interestingly, 2DOG also reduced the apparent molecular weight of TF to ~40 kDa. As a comparison blocking glycosylation with tunicamycin (4 µg/mL) also reduced the amount of TF produced and reduced its molecular weight further (~36 kDa). The activity of TF in the conditioned medium was significantly reduced by 2DOG or tunicamycin (Figure 3C). Taken together, these results further demonstrate that glycolysis is necessary for TF glycosylation and 2F-induced TF synthesis. At the concentrations used, 2DOG and tunicamycin did not induce LDH release. LDH levels were less than 6% of the cellular totals levels all experiments. See Supplementary Figure S1.

## 3. Discussion

CKD affects 8–16% of the world’s population and this number is projected to increase in the years to come as the incidence of its major risk factors, diabetes, hypertension and obesity, continue to rise [27]. The leading cause of CKD is diabetic nephropathy. In this cohort, poorly regulated hyperglycemia has been shown to significantly enhance dyslipidemia, systemic inflammation, microvascular damage and coagulation [1,2,6,27,28]. Hyperglycemia also directly damages kidney tubular epithelial cells which have an essential role in glucose homeostasis through gluconeogenesis and reabsorption of filtered glucose [29,30,31,32,33]. The coagulopathies common in diabetes and CKD are not clearly understood. Non-specific therapies targeting these clotting disorders, such as anti-thrombotic and anti-platelet agents, have been largely ineffective due to bleeding complications [9].

Studies using animal models of kidney disease have indicated a pathological role for PAR2 in CKD. PAR2 knockout (PAR-2^(−/−)^) mice are protected to some extent from fibrosis induced by unilateral ureteral obstruction, an adenine-induced CKD, cisplatin nephrotoxicity or experimental glomerulonephritis [19,34,35,36]. Deposition of collagen and fibrin, elevated expression of alpha smooth muscle actin and TF and expression of other indicators of nephrotoxicity were reduced. In a model of crescentic glomerulonephritis deposition of fibrin and expression of plasminogen activator inhibitor are reduced in PAR-2^−/−^ mice [36]. Further to this pharmacologically blocking PAR2 also reduce glomerular crescent formation and glomerular fibrin deposition [23]. A role for PAR2 in diabetic kidney disease has also been reported [37]. As in vivo PAR2 is activated by various protease it is suggested that targeting specific PAR2-activating proteases or PAR2 itself may be of therapeutic value.

The key findings of this study were that (I) a PAR2-activating peptide agonist induced TF synthesis in HTECs; (II) culturing these cells in a medium that mirrors a diabetic concentration of glucose (25 mM) significantly enhanced PAR2-induced TF protein synthesis and secretion; (III) PAR2 activation leads to a significant increase in glucose consumption and lactate production; and (IV) a specific PAR2 antagonist, glycolysis inhibitor, or N-linked glycosylation inhibitor, each significantly reduced PAR2-mediated TF production.

PAR2 is highly expressed in HTECs and its activation elicits a potent pro-inflammatory response characterized by secretion of cytokines, including TNFα, CSF-2 and MCP-1 [38,39,40,41]. In previous studies, a marked increase in TF mRNA expression and TF protein synthesis was seen in these cells when they were treated with a potent PAR2-activating peptide, 2F or PAR2-activating proteases [38,42]. The present study now shows that PAR2-induced TF expression is glucose availability/glycolysis dependent. Only a small portion of the TF produced in response to 2F was released by these cells. As TF is an integral membrane protein, it is not readily released from cells and when it is it is incorporated onto the surface of extracellular vesicles (EV) [42,43]. TF bound in this way was shown to be active using by a factor Xa-generating assay.

The enhanced expression of TF by cells is likely to further increase PAR2 activation as TF represents an important co-factor for factor Xa-PAR2 and factor VIIa-PAR2 activation. As membrane barriers (vascular barriers included) are compromised in response to injury or infection, the access of cells to circulating clotting proteins is enhanced. Inflammatory cytokines are known to upregulate PAR2 and TF expression in cells and so are likely to promote local PAR2 responses, which include inflammation, immune activation, and metabolic changes [44,45]. A previous study has demonstrated that higher levels of glucose enhance TF synthesis by endothelial cells in response to thrombin [46]. Thus, high levels of glucose could potentially enhance TF synthesis by PAR1 and PAR2 on cells that express both receptors. The PAR2-induced expression of TF by HTECs represents another layer of complexity to the coagulation/inflammatory response by cells. The enhanced glucose consumption and lactate production observed in this study suggest that metabolic changes are associated with PAR2 activation not unlike metabolic changes seen in immune cells when they are activated [47]. The metabolic adaption of cells to injury and infection plays an integral role in innate immune responses [48].

Although a role for PAR2 in kidney (patho)-physiology is not clear, its reported links to ion channel activity, epithelial barrier integrity, blood flow, inflammation and fibrosis suggest potential roles [20]. Questions remain as to which proteases are relevant in vivo. Possibilities include proteases released from activated, injured or damaged cells (inflammatory or epithelial), proteases produced and released from mast cells (tryptase or chymase), circulatory coagulation proteases that enter tissues when the vascular permeability is compromised (factors Xa or VIIa), and epithelial proteases (trypsin(ogen)s, matriptase, kallekreins) that are expressed in the kidney epithelial cells and under certain circumstances can activate PAR2 [18,49].

Healthy humans have a tightly regulated blood glucose concentration that normally only very briefly rises above 6.0 mM. Higher glucose concentrations, such as that examined here in vitro (25 mM), are often used to simulate an uncontrolled diabetic milieu. This high glucose concentration produces a range of physiological changes associated with diseases in vivo, including enhanced rates of cell growth, protein glycation, free radical production, and cellular lipid peroxidation. Any of these events could involve altered activity of enzymes or other signaling proteins potentially associated with increased PAR2 expression or activation. Hyperglycemia in vivo is likely to elicit similar responses on kidney cells as those examined here in vitro. As adding higher levels of D-glucose to cells in culture may have an osmotic effect we used L-glucose as a control to simulate this potential osmotic effect. Adding increased amounts of L-glucose did not replicate the actions of elevate D-glucose concentrations.

The metabolic inhibitor 2DOG was shown here to significantly reduce PAR2-induced TF synthesis. 2DOG is actively taken up by the glucose transporters and phosphorylated but, since it cannot be metabolized, 2-DOG-6-phosphate accumulates and inhibits phosphor-6-glucose isomerase and hexokinase [50]. In addition, 2DOG affects N-glycosylation, which is highly dependent on catabolic glucose intermediates. TF has four potential glycosylation sites, three on the external N terminus and one on the internal C terminus [12,15]. As the fully glycosylated molecular weight of HTEC TF is 45 kDa, all the glycosylation sites are likely to be occupied. A molecular weight of ~40 kDa produced by addition of 5 mM 2DOG suggests that TF retains some post-translational glycosylation. Whether some N-linked glycosylation sites are fully glycosylated, and others are not, or whether all glycosylation sites have modified sugar moieties, is not clear. Further studies could be performed with varying concentrations of 2DOG and glycomics. The reduction in molecular weight of TF when 2DOG is added indicates the importance of glycolysis for PAR2-induced synthesis of functionally mature TF protein. The molecular weight of other glycosylated proteins such as fibronectin (4% of its molecular weight is due to glycosylation), appear not to be altered by 2DOG (data not shown).

Despite the discussion above on the action of 2DOG on TF glycosylation, without further investigations, it is not possible to eliminate other events triggered by 2DOG including changes in protein synthesis. Beyond its direct effect on glycolysis, there is likely to be a deficit in cellular ATP as metabolites that feed into the Krebs cycle are reduced. This would ultimately reduce cell viability and glucose levels within the cell and medium may remain unchanged. However, over a 24 h experimental period, 2DOG at 5 mM did not alter LDH release by the cells (remaining below 6% of total cellular LDH), suggesting that the cells remained viable. We also cannot rule out the possibility that cleavage of mature TF is promoted by 2DOG and that this is responsible for the molecular weight changes observed in TF observed.

Another fact to consider is that signaling via PAR2 can be altered by the extent of PAR2 glycosylation. The glycosylation of PAR2 is known to limit the access of the proteases tryptase to the PAR2 activation cleavage sites [51].

Tunicamycin is often used to block N-linked glycosylation and, when added to HTECs in the presence of 25 mM glucose and 2F, it significantly reduced the molecular weight of TF (~36 kDa) as well as the amount of TF produced. A molecular weight for TF of ~36 kDa suggests that TF has been synthesized without any N-linked glycosylation [15].

## 4. Materials and Methods

### 4.1. Materials

The PAR2 antagonist, I-191, and PAR2 peptide agonist, 2f-LIGRLO-NH_2_ (2F), were synthesized and purified by the Institute for Molecular Bioscience, The University of Queensland as previously described [52,53]. The human TF antibody from R&D Systems (Cat #AF2339) was used at a dilution of 1/2000. The horseradish peroxidase (HRP)-linked secondary antibodies were also from R&D Systems and were used at ≥1/30,000 dilution. The base growth media used were Gibco Dulbecco’s Modified Eagle’s Medium (DMEM)/Ham’s F12 (Cat #11320) and Gibco DMEM (Cat #11995) (ThermoFisher Scientific, Mt Waverley, VIC, Australia). Tunicamycin and 2-deoxyglucose were from Sigma-Aldrich (St. Louis, MO, USA).

### 4.2. Tubule Cell Isolation and Cell Culture

Segments of macroscopically and histologically normal kidney cortex (~10 g) were obtained aseptically from the non-cancerous pole of adult human kidneys removed surgically because of small kidney tumors. Patients were otherwise healthy. Informed consent was obtained prior to each operative procedure and the use of human kidney tissue for primary cell culture was approved by the Princess Alexandra Hospital Research Ethics Committee, Brisbane, Australia (ethics number: HREC/12/QPAH/125). The method for isolation and culture of human kidney tubular epithelial cells (HTECs) is described in detail elsewhere [54]. Following isolation, cells were cultured in a serum-free, hormonally defined DMEM/F12 medium containing epidermal growth factor (20 ng/mL), insulin (5 µg/mL), transferrin (5 µg/mL), hydrocortisone (50 nM), triiodothyronine (5 pM), selenium (50 nM), penicillin (50 U/mL), and streptomycin (50 µg/mL). Cells were routinely cultured in this medium. At least 30 donor cell isolates were used in this study.

### 4.3. Cell Treatments

All experiments were performed on confluent passage 1 or 2 HTECs cultured in 48-, 12- or 6-well plates (Corning, NY, USA). Before experimentation, cells were made quiescent by two washes followed by incubation for 24 h in basic media (DMEM medium with antibiotics). In experiments where the glucose concentration was modulated to 5 or 25 mM, the basic medium used contained 5 mM glucose. At 24 h, the medium was changed to 0 or 25 mM glucose. In some experiments, the medium was supplement of L-glucose at an equivalent concentration of D-glucose to control for the osmotic effects of various D-glucose concentrations added to cells. Effects of PAR2 activation using peptide, 2F on TF production were measured by Western blotting, a TF ELISA and TF activity assay. Effects of PAR2 activation using peptide, 2F on TF production were measured by Western blotting, a TF ELISA and TF activity assay. The cells and cell conditioned media were harvested according to routine techniques and, if necessary, stored at −80 °C until required for analysis. Harvested medium was centrifuged at 900 xg prior to storage. In some cases, the conditioned medium from the cells was concentrated 10-fold using a Nanosep Centrifugal Device with a 3 kDa (OD003C34) molecular weight cut off (Pall, Melbourne, VIC, Australia). In some experiments, 2-deoxyglucose (2DOG, 5 mM) or tunicamycin (4 µg/mL) was added to cultures. To assess if 2DOG or tunicamycin were toxic to cells lactate dehydrogenase (LDH) released into the culture medium was measured using The Pierce™ LDH Cytotoxicity Assay Kit (ThermoFisher Scientific Catalog number:88953, Freemont, CA, USA) was used according to manufacturer’s protocol (see Supplementary Figure S1).

### 4.4. Western Blot Analysis

For Western blot analysis, the cells were grown to confluence in 6-well plates. A volume of 2 mL of medium was used per well. At the end of the experiment, the medium was harvested, centrifuged at 900 xg and stored at −80 °C. Cells were washed twice with ice-cold phosphate-buffered saline (PBS) and lysed with 150 µL of radioimmunoprecipitation assay (RIPA) buffer (Sigma-Aldrich, Castle Hill, NSW, Australia, Cat # R0278), containing a protease inhibitor cocktail (Sigma-Aldrich, Castle Hill, NSW, Australia, Cat. # P8340). Cells were further disrupted by sonication, cell debris pelleted by centrifugation (13,000× *g*, 20 min), and the supernatant collected. The total protein concentrations were measured using the BCA kit from ThermoFisher Scientific, (Mt Waverley, VIC, Australia). Equal amounts of concentrated, conditioned medium or cell protein (20–30 µg) were diluted in Bolt LDS sample buffer containing 50 mM dithiothreitol, heated to 70 °C for 10 min, separated on a 4–12% NuPAGE gel (ThermoFisher Scientific, Mt Waverley, VIC, Australia) and electro-transferred to a 0.4 µm polyvinylidene difluoride membrane (Thermo Fisher Scientific, Mt Waverley, VIC, Australia). Membranes were blocked with SuperBlock™ (PBST, ThermoFisher Scientific, Mt Waverley, VIC, Australia) for 1 h before incubation overnight with the primary antibody diluted in blocking buffer. After washing four times, 5 min each, with wash buffer (PBS containing 0.05% Tween-20), the appropriate secondary HRP-conjugated antibody (R&D Systems, Minneapolis, MN, USA) in blocking buffer was added to the membranes for a 40 min incubation at room temperature (RT) with gentle agitation. Membranes were washed as above before development with SuperSignal West Pico Plus, or SuperSignal West Femto (ThermoFisher Scientific, Mt Waverley, VIC, Australia). A Bio Rad ChemiDoc MP Imaging System was used to capture images. Arcsoft Photo studio 5 was used to create images for this document. The image size, brightness and contrast were adjusted using this software. ImageJ (NIH, Bethesda, MD, USA) was used to estimate band intensity on some images. Pictures are representative images from at least three independent experiments. To control for equal protein loading, some membranes were re-probed with a pan-actin monoclonal antibody (1:5000, Cat No. ACTN05C4, ThermoFisher Scientific, Freemont, CA, USA) or beta-tubulin monoclonal (1/5000, Cat No. T4026, Sigma-Aldrich, Castle Hill, NSW, Australia) diluted in blocking buffer overnight. Following washing, the secondary antibody was used at a dilution of ≥1:30,000. The actin and tubulin bands were visualized as above. A Ponceau red stain was also used to assess protein transfer and loading.

### 4.5. Tissue Factor ELISA

TF was measured in the HTEC lysate and conditioned culture medium using a specific DuoSet enzyme-linked immunosorbent assay (ELISA) kit (R&D Systems, Minneapolis, MN, USA) according to the manufacturer’s protocols. For efficient detection of TF in the conditioned medium by ELISA, it was necessary to incubate the medium with a non-ionic detergent, β-octyl-glucopyranoside (OG). This was used at a final concentration of 15 mM with a 1 h incubation at RT. Samples were finally briefly sonicated and diluted further in 1% BSA in PBS prior to use in the ELISA.

### 4.6. Tissue Factor Activity Assay

A human TF chromogenic activity assay was adapted from a previously published method (Lwaleed et al., 2000). Active TF standards ranging from 7.8 to 250 pM were from Abcam (Cambridge, UK). The reaction mix consisted of 10 µL of sample, 5 µL of recombinant FVIIa (final concentration of 10 nM; NovoSevenRT, Novo Nordisk), 10 µL of Factor X (final concentration of 100 nM, Haematologic Technologies Inc. Essex Junction VT, USA), 10 µL of CaCl_2_ (final concentration of l0 mM) and 45 µL of Tris-buffered saline. After 1 h incubation at RT, the reaction was initiated by adding 10 µL of Factor Xa substrate (Chromogenix S-2222). Readings at 405 nM were taken at 1 min intervals, with mixing for 2 h using a Multiskan FC Microplate reader (ThermoFisher Scientific, Freemont, CA, USA).

### 4.7. Glucose and Lactate Concentrations

The glucose and lactate concentrations of the cell culture medium were measured with a Beckman DxC800 general Chemistry analyzer (Beckman Coulter, Brea, CA, USA).

### 4.8. Statistical Analysis

All studies were performed in at least triplicate from HTEC cultures obtained from at least three separate human donors unless otherwise indicated. Each experiment contained internal controls originating from the same culture preparation. In some cases, for the purposes of analysis, each experimental result was expressed as a change from the maximal response, which was regarded as 100, and analyzed independently. Results were expressed as the mean ± standard deviation (SD). GraphPad Prism version 6 was used to construct graphs and for statistical analysis. *P* values ≤ 0.05 were considered significant. T-test and/or ANOVA were used to assess statistical difference between treatments.

## 5. Conclusions

We have shown that PAR2 activation induces synthesis and secretion of tissue factor by HTECs, which is significantly enhanced by a high, diabetic-like, glucose concentration. An antagonist of PAR2 (I-191), an inhibitor of glycolysis (2DOG) or an inhibitor of glycosylation (tunicamycin) was able to significantly reduce PAR2-induced TF synthesis and secretion. These findings may help explain how elevated glucose concentrations promote clotting abnormities in DKD and suggest that a PAR2 antagonist might be therapeutically useful in limiting adverse clotting events in DKD.

## 6. Patent

D.P.F. is an inventor on a patent AU20109033378 covering PAR2 agonists and antagonists that is owned by The University of Queensland.

## Data Availability

Additional data supporting the reported results of this article are available upon request from the corresponding author. The data are not publicly available due to privacy.

## Supplementary Materials

The following are available online at https://www.mdpi.com/article/10.3390/ijms22147532/s1.
