# Supplementary material for: PAR2-Induced Tissue Factor Synthesis by Primary Cultures of Human Kidney Tubular Epithelial Cells Is Modified by Glucose Availability
Source: Int J Mol Sci. 2021 Jul 14;22(14):7532. doi: 10.3390/ijms22147532 (PMC8304776; doi:10.3390/ijms22147532)
Supplement: Supplementary file 1 [file ijms-22-07532-s001.zip › ijms-1268098-supplementary.pdf]

Figure S1

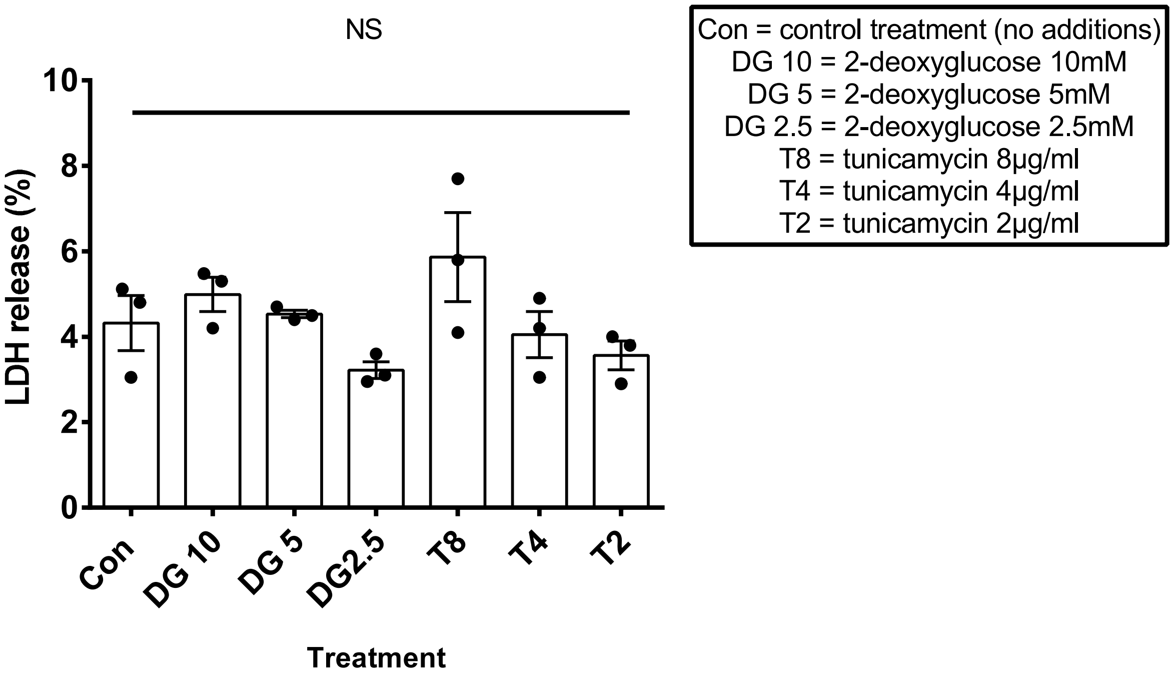

Supplementary Figure S1. Lactate dehydrogenase (LDH) release by HTEC treated with 2DOG or tunicamycin at the indicated concentrations was measured to test for toxicity.

The Pierce™ LDH Cytotoxicity Assay Kit (Catalog number:88953) (ThermoFisher Scientific, Freemont, CA, USA) was used according to manufacturers protocol was followed
